# Low dose angiostatic treatment counteracts radiotherapy-induced tumor perfusion and enhances the anti-tumor effect

**DOI:** 10.18632/oncotarget.12814

**Published:** 2016-10-21

**Authors:** Esther A. Kleibeuker, Emmanouil Fokas, Philip D. Allen, Veerle Kersemans, Arjan W. Griffioen, John Beech, Jaehong H. Im, Sean C. Smart, Kitty C. Castricum, Jaap van den Berg, Iris A. Schulkens, Sally A. Hill, Adrian L. Harris, Ben J. Slotman, Henk M. Verheul, Ruth J. Muschel, Victor L. Thijssen

**Affiliations:** ^1^ Department of Radiation Oncology, VU University Medical Centre, De Boelelaan, HV Amsterdam, The Netherlands; ^2^ Department of Medical Oncology, VU University Medical Centre, De Boelelaan, HV Amsterdam, The Netherlands; ^3^ Oxford Institute for Radiation Oncology and Biology, University of Oxford, Oxford, UK; ^4^ Department of Molecular Oncology, University of Oxford, Oxford, UK

**Keywords:** cancer, radiotherapy, angiogenesis, tumor perfusion, combination therapy

## Abstract

The extent of tumor oxygenation is an important factor contributing to the efficacy of radiation therapy (RTx). Interestingly, several preclinical studies have shown benefit of combining RTx with drugs that inhibit tumor blood vessel growth, i.e. angiostatic therapy. Recent findings show that proper scheduling of both treatment modalities allows dose reduction of angiostatic drugs without affecting therapeutic efficacy. We found that whilst low dose sunitinib (20 mg/kg/day) did not affect the growth of xenograft HT29 colon carcinoma tumors in nude mice, the combination with either single dose RTx (1x 5Gy) or fractionated RTx (5x 2Gy/week, up to 3 weeks) substantially hampered tumor growth compared to either RTx treatment alone. To better understand the interaction between RTx and low dose angiostatic therapy, we explored the effects of RTx on tumor angiogenesis and tissue perfusion. DCE-MRI analyses revealed that fractionated RTx resulted in enhanced perfusion after two weeks of treatment. This mainly occurred in the center of the tumor and was accompanied by increased tissue viability and decreased hypoxia. These effects were accompanied by increased expression of the pro-angiogenic growth factors VEGF and PlGF. DCE-MRI and contrast enhanced ultrasonography showed that the increase in perfusion and tissue viability was counteracted by low-dose sunitinib. Overall, these data give insight in the dynamics of tumor perfusion during conventional 2 Gy fractionated RTx and provide a rationale to combine low dose angiostatic drugs with RTx both in the palliative as well as in the curative setting.

## INTRODUCTION

Tumor oxygenation is an important predictor of sensitivity to radiation therapy (RTx) [1, 2]. Surprisingly, several pre-clinical studies and clinical trials have shown a potential benefit of combining RTx with angiostatic treatment, i.e. inhibition of blood vessel formation [3-7]. This has partly been attributed to a transient improvement of tumor oxygenation due to vascular normalization in response to angiostatic drugs as observerd in *in vivo* tumor models [8-12]. However, evidence supporting such a response in patients is scarce [13, 14] and it is unknown for how long the normalisation would last in patients. The temporary character of improved oxygenation suggests only a limited effect of vascular normalization which would not benefit patients receiving conventional 2 Gy fractionated RTx (RTx^FR^) for several weeks. In addition, our previous preclinical observations and several clinical case reports by others show that maintenance angiostatic therapy during and after RTx is also beneficial [15-17]. This demonstrates that there are other feasible treatment schedules of the combination therapy.

The efficacy of angiostatic therapy during RTx has also been attributed to the angiogenic rebound effect, i.e. the induction of angiogenic growth factor expression by RTx. Indeed, several reports using different tumor models have shown that RTx can induce the expression of e.g. VEGF, FGF2 (bFGF) and PDGF [3, 18-24]. In line with this, both single dose RTx (RTx^SD^) as well as RTx^FR^ are known to affect tumor perfusion and oxygenation which appear to be dependent on dosing and scheduling [25-31]. This provides opportunities to optimize the combination of RTx with angiostatic therapy. For example, we have recently shown that optimal scheduling of RTx^SD^ combined with angiostatic therapy allows dose reduction of the angiostatic drug without affecting therapeutic outcome [17]. This is clinically relevant as dose reductions could reduce toxicities that are observed when RTx is combined with angiogenesis inhibitors [14, 17]. Whether dose reductions can also be applied when angiostatic treatment is combined with RTx^FR^ is not known. To better understand the interaction between RTx and angiostatic therapy we investigated the effects of RTx^SD^ and RTx^FR^ in combination with low dose angiostatic treatment on tumor growth and tumor perfusion.

## RESULTS

### Low dose sunitinib after RTx enhances anti-tumor efficacy

**Figure 1:**
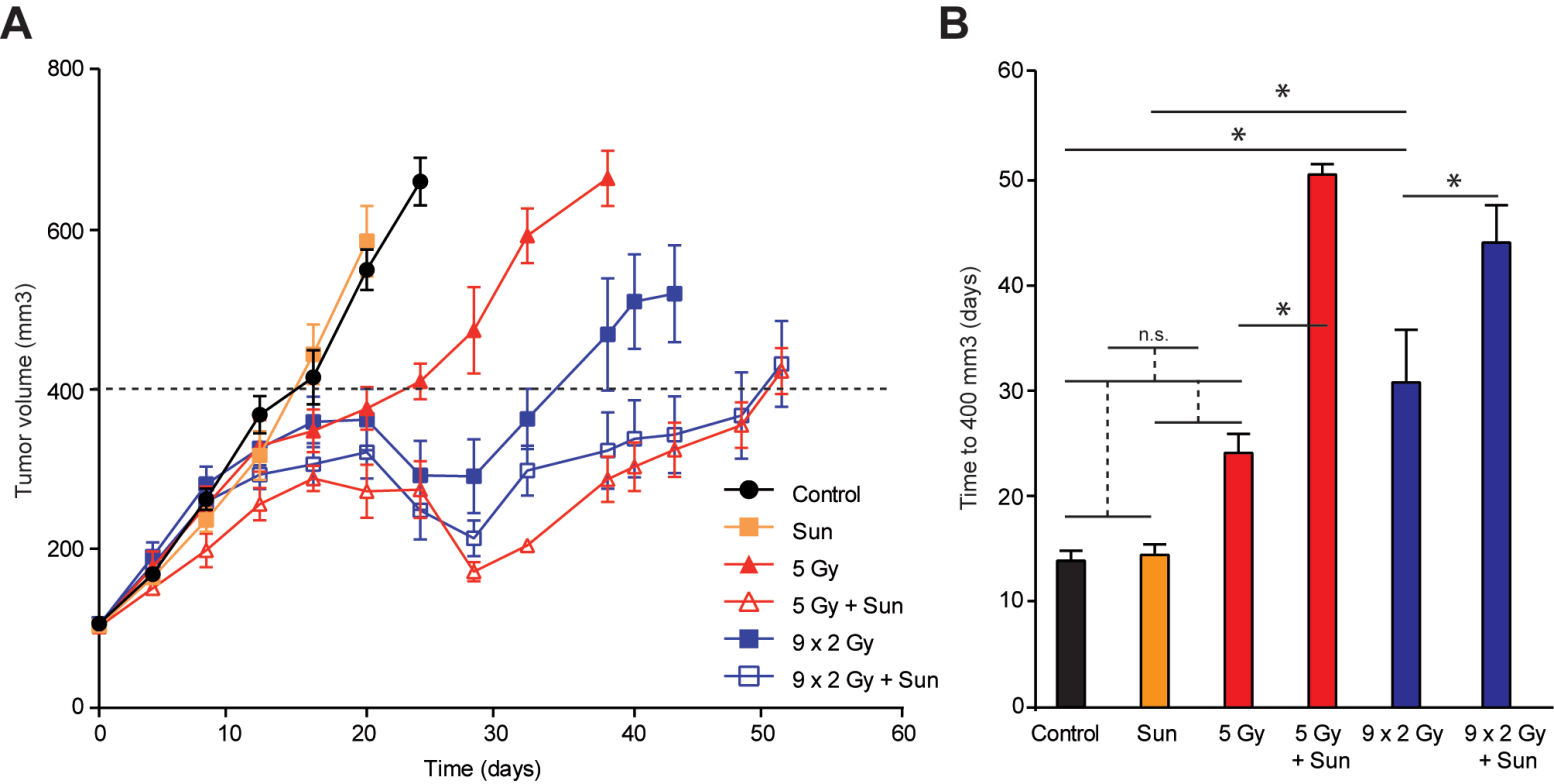
HT29 tumor growth in balb/c nude mice, treated with radiation therapy (RTx) and low dose sunitinib **A.** Tumor growth curves of HT29 xenograft tumors in balb/c nude mice. Tumors were grown to 100 mm^3^ and subsequently treated as indicated. RTx consisted of daily 2 Gy fractions (5/week) or a single dose of 5 Gy. Sunitinib was daily administered by oral gavage (20 mg/kg/day). In case of combination therapy, sunitinib treatment was started after the first dose of RTx and continued until the end of the experiment. Data are shown as average +/− SEM. N = 7-10 per experimental group. **B.** Bar graph showing the time for tumors to grow to 400 mm^3^. * *p* < 0.05 (Student *t*-test). Data are shown as average +/− SEM. N = 7-10 per experimental group.

We have previously shown that low dose sunitinib given after RTx^SD^ induces a more pronounced anti-tumor effect than sunitinib applied prior to RTx [17]. To explore the effect of low dose sunitinib on RTx^FR^, nude mice with xenograft tumors of colorectal adenocarcinoma cells (HT29) were treated with either RTx^SD^ (1x 5 Gy) or RTx^FR^ (2 Gy/day, 5 days/week) for two weeks, with or without sunitinib. In case of combination therapy, low dose sunitinib (20 mg/kg/day) was applied daily after the start of RTx. Low dose sunitinib did not affect tumor growth in itself. RTx^FR^ caused a longer tumor growth delay than RTx^SD^ alone (Figure 1A and 1B). Combining either RTx^SD^ or RTx^FR^ with low dose sunitinib extended the tumor growth delay significantly compared to both RTx regimens alone (Figure 1A and 1B). The growth reduction by sunitinib was most prominent after RTx^SD^. No toxicities were noted during the experiments (Supplementary Figure S1). Together, these data confirm previous results, demonstrating that low dose sunitinib significantly enhances the anti-tumor effect of RTx.

### Radiation therapy enhances tumor perfusion and reduces tumor hypoxia

**Figure 2:**
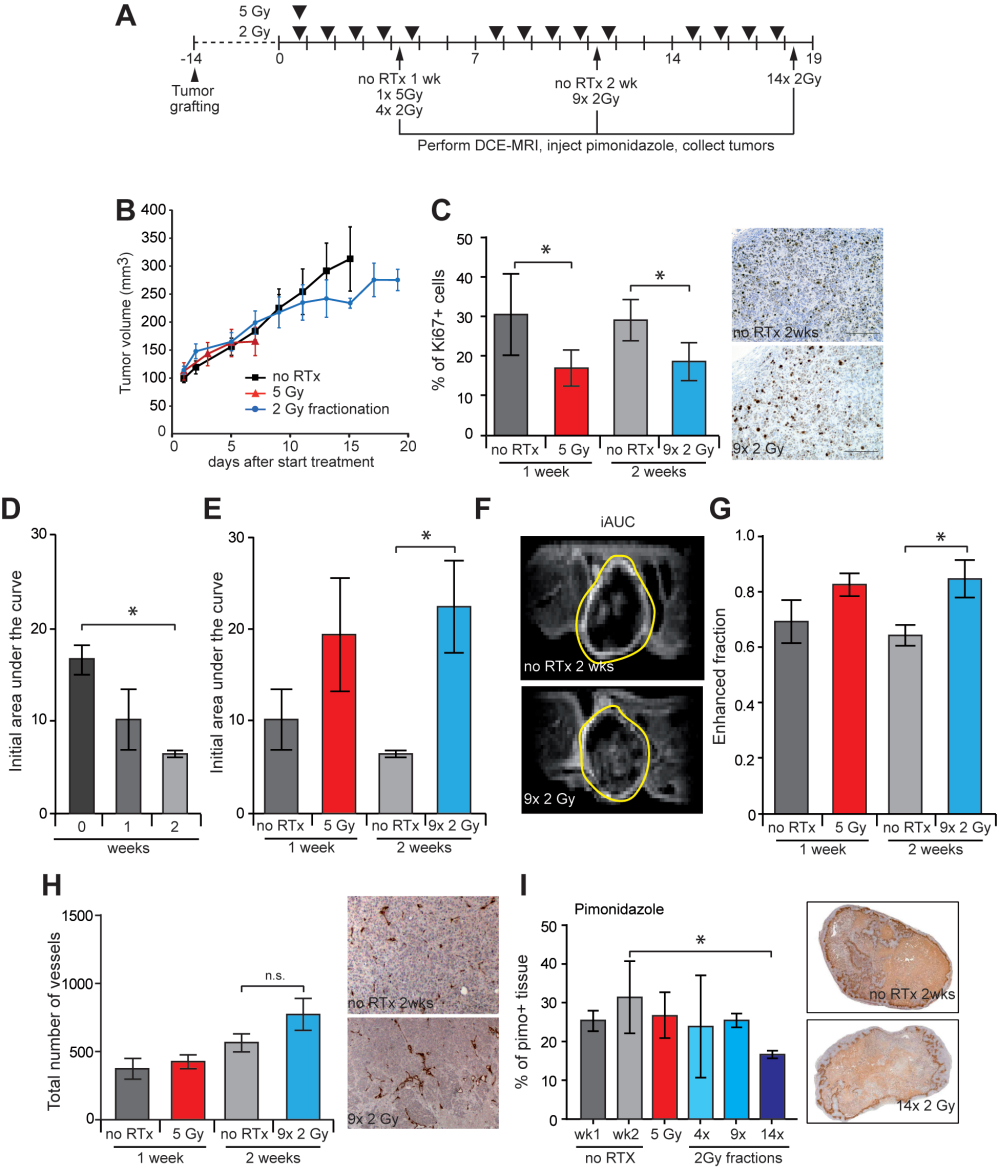
Effect of RTx on tumor perfusion and vasculature in HT29 xenograft tumors **A.** Overview of treatment schedules, dynamic contrast enhanced (DCE) MRI time points and tumor harvesting with the HT29 xenografts in balb/c nude mice. **B.** Tumor growth curves of HT29 xenograft. Tumors were grown to 100 mm^3^ and subsequently treated as indicated. Single dose RTx consisted of 1x 5 Gy and fractionated RTx consisted of daily 2 Gy fractions (5/week) for 1, 2, or 3 weeks. For each treatment group receiving FR RTx, the final fractions were omitted, due to protocol restrictions. **C.** The proliferation index of the HT29 tumors was determined by IHC staining of Ki67 (brown). **D.** Initial area under the curve determined by DCE-MRI, of tumors that did not receive RTx treatment. **E.** The initial area under the curve (iAUC) of each tumor as determined by DCE-MRI. **F.** Representative image of a DCE-MRI scan of a tumor performed at the end of the second treatment week. The image displays the cumulative enhancement after contrast infusion. The tumor boundary is indicated by the yellow line. **G.** The fraction of enhanced voxels during the scan as determined by DCE-MRI. **H.** Total number of blood vessels in the viable tissue was measured with IHC staining of CD31 (dark brown). **I.** The percentage of hypoxic tissue within the viable tissue as determined by pimonidazole staining (brown). Pimonidazole was i.v. injected before sacrificing the mouse. Tumor growth data are shown as average +/− SEM. All data are shown as average +/− SD. N = 4-5 per experimental group. * *p* < 0.05 (Mann-Whitney U test).

To explore the mechanisms by which RTx sensitizes the tumor to low dose sunitinib, tumor perfusion and hypoxia were examined. To that end, established HT29 tumors in nude mice were locally treated with either RTx^SD^ (1x 5 Gy) or RTx^FR^ (2 Gy/day, 5 days/week) for up to 3 weeks. Tumor perfusion was determined at the end of each treatment week by dynamic contrast enhanced magnetic resonance imaging (DCE-MRI). Tumors were harvested weekly for further analyses (Figure 2A). Tumor volume measurements showed similar growth delays as in Figure 1 (Figure 2B). Consistent with the tumor growth delay, there was significant reduction in tumor cell proliferation 1 week after RTx^SD^, as well as after 2 or 3 weeks of RTx^FR^ as measured by Ki67 immunohistochemistry (Figure 2C, Supplementary Figure S2).

DCE-MRI in non-treated tumors revealed a significant decrease in tumor perfusion, measured by the initial area under the curve (iAUC at 150 seconds after Gadolinium injection) in time as the tumor grew (Figure 2D). One week after either RTx^SD^ or RTx^FR^ the iAUC was not significantly affected although a trend towards increased perfusion was noticeable (Figure 2E, Supplementary Figure S2). In line with this, after 2 weeks of RTx^FR^ there was a > 3 fold increase in the average iAUC (Figure 2E and 2F, Supplementary Figure S2). This was not explained by differences in tumor growth as both untreated and treated tumor volumes were similar. Enhancement of the fraction of voxels assessed by DCE-MRI (Figure 2G, Supplementary Figure S2), as well as a trend towards an increase in the number of tumor vessels (Figure 2H, Supplementary Figure S2) and a decrease in tumor hypoxia (Figure 2I + Supplementary Figure S2) were indicative for an improvement of tumor perfusion during RTx^FR^.

### RTx enhances cancer cell repopulation in the tumor core

**Figure 3:**
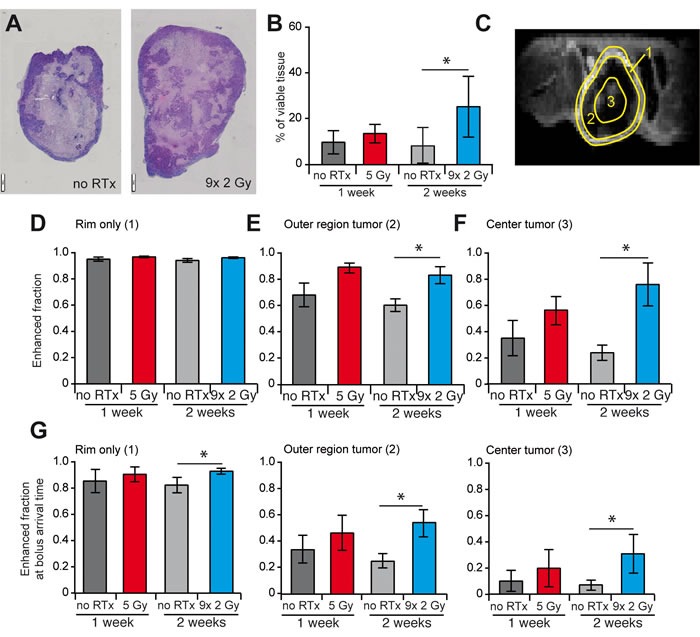
Effect of RTx on vasculature and tissue viability and perfusion in different regions of the tumor **A.** Representative images H/E staining of a non-irradiated tumor (left) and a tumor that received 9x 2 Gy (right). Different tumor sizes are due to different section planes of non-spherical tumors, i.e. lateral *vs*. longitudinal. **B.** The percentage of viable tissue in the center of the tumor, determined with H/E staining. **C.** Representative image of DCE-MRI showing the division of the tumor into three regions, i.e. the tumor rim (1), the tumor outer region (2) and the tumor center (3). **D.**-**F.**Enhanced fraction of voxels in the three different regions of the tumor as determined by DCE-MRI. All data are shown as average +/− SD. N = 4-5 per experimental group. * *p* < 0.05 (Mann-Whitney U test).

To evaluate the effects of enhanced perfusion, the amount of viable tissue was evaluated with H/E staining. A significant increase of viable tissue was observed in the center of the tumor after 2 weeks of RTx^FR^ while a decrease in overall tissue necrosis was observed (Figure 3A and 3B, Supplementary Figure S3). The percentage of viable tissue significantly correlated with the amount of vascular CD31 staining (Figure 3C). Since these findings suggest an increased effect on tumor cell repopulation in the tumor core of RTx^FR^ treated tumors, we analyzed tissue perfusion in different regions of the tumor, i.e. the rim, the outer region and the center (Figure 3D). This revealed that the tumor rim was always well perfused, regardless of the different RTx schedules (Figure 3E, Supplementary Figure S3). While induction of perfusion after RTx was noted in the outer region of the tumor, the largest increase was observed in the center of the tumor, reaching statistical significance after 2 weeks RTx^FR^ (Figure 3F and 3G, Supplementary Figure S3). An increase was also observed for voxels that enhanced on the first time point after injection (bolus arrival time (BAT)) confirming that the effect could be attributed to local perfusion rather than diffusion from neighboring regions (Supplementary Figure S3). Overall, the described results further indicate that RTx facilitates a better vascularization/perfusion of the tumor core.

### RTx induces a pro-angiogenic response in tumor cells

**Figure 4:**
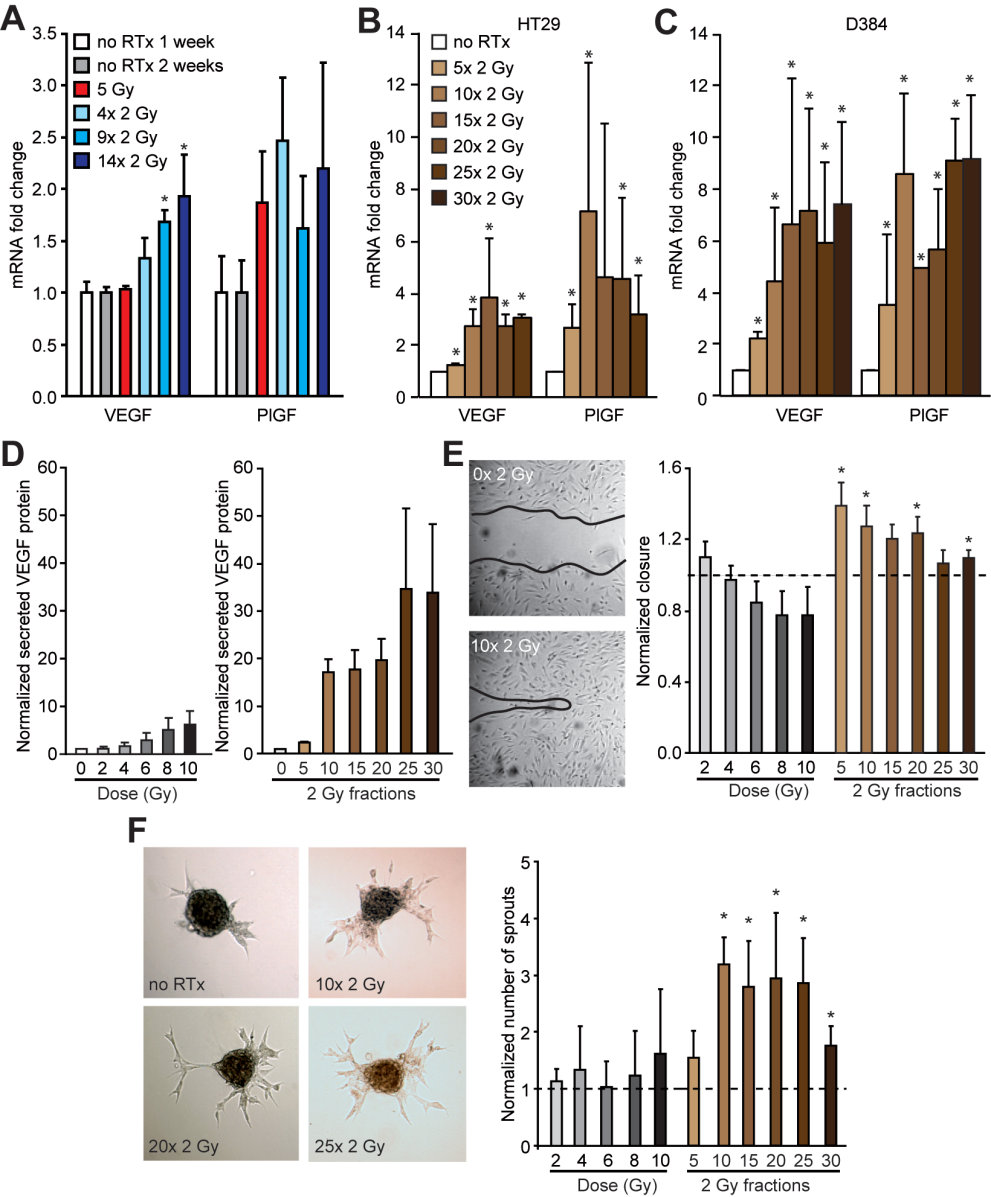
Enhanced pro-angiogenic growth factor expression *in vivo* and *in vitro* after RTx in cancer cells **A.** Relative mRNA expression of the pro-angiogenic growth factors VEGF and PlGF in HT29 xenograft tumors in balb/c nude mice after RTx. N = 4-5 per experimental group. **B.** Relative mRNA expression of the pro-angiogenic growth factors VEGF and PlGF in HT29 cells *in vitro* after FR RTx. N = 3. **C.** Similar as in B for D384 cells. N = 3. **D.** Normalized secreted VEGF protein expression in the supernatant of HT29 cells after single dose or FR RTx. The protein expression was normalized to the number of cells. N = 3. **E.** Migration assay with endothelial cells (HUVECs) with conditioned medium of irradiated HT29 cells. The width of the scratch was normalized to non-irradiated condition (dotted line). N = 2-3 individual HUVEC batches for each batch of conditioned medium (N = 3). * *p* < 0.05 *vs*. no RTx (Mann Whitney rank sum test). **F.** Sprouting assay with HUVEC spheroids with conditioned medium of irradiated HT29 cells. The number of sprouts was normalized to non-irradiated condition (dotted line). N = 2-3 individual HUVEC batches for each batch of conditioned medium (N = 3). * *p* < 0.05 *vs*. no RTx (Mann-Whitney U test). All data are shown as average +/− SD.

To explore how RTx could enhance tumor perfusion, the mRNA expression levels of prominent pro-angiogenic growth factors was determined. An induction of vascular endothelial growth factor (VEGF) and placental growth factor (PlGF) expression in response to RTx^FR^ was measured in HT29 tumors and cultured HT29 cells (Figure 4A + 4B, Supplementary Figure S4A + S4B). This induction was generalizable as it was also observe in D384 glioblastoma cells received fractionated irradiation (Figure 4C, Supplementary Figure S4C). Furthermore, in line with the enhanced mRNA levels, the secretion of the VEGF protein *in vitro* was enhanced after RTx in a dose-dependent fashion in both HT29 and D384 cells (Figure 4D, Supplementary Figure S4D-S4F). To confirm the functional relevance of the RTx-induced pro-angiogenic response, human umbilical vein endothelial cells (HUVEC) were cultured in the presence of conditioned medium from irradiated cancer cells. The conditioned medium resulted in a pro-angiogenic phenotype as evidenced by enhanced migration and sprouting of the endothelial cells (Figure 4E-4F, Supplementary Figure S4G-S4H). The effects were most pronounced in conditioned medium collected after RTx^FR^. Of note, for both HT29 and D384 cells *in vitro*, surviving colonies were observed after 30x 2 Gy (0.18% and 0.64% fraction of surviving colonies as determined by colony formation assay, respectively). Collectively, these data demonstrate the angiostimulatory potential of tumor cells surviving RTx.

### Low dose sunitinib counteracts RTx-induced tumor perfusion

**Figure 5:**
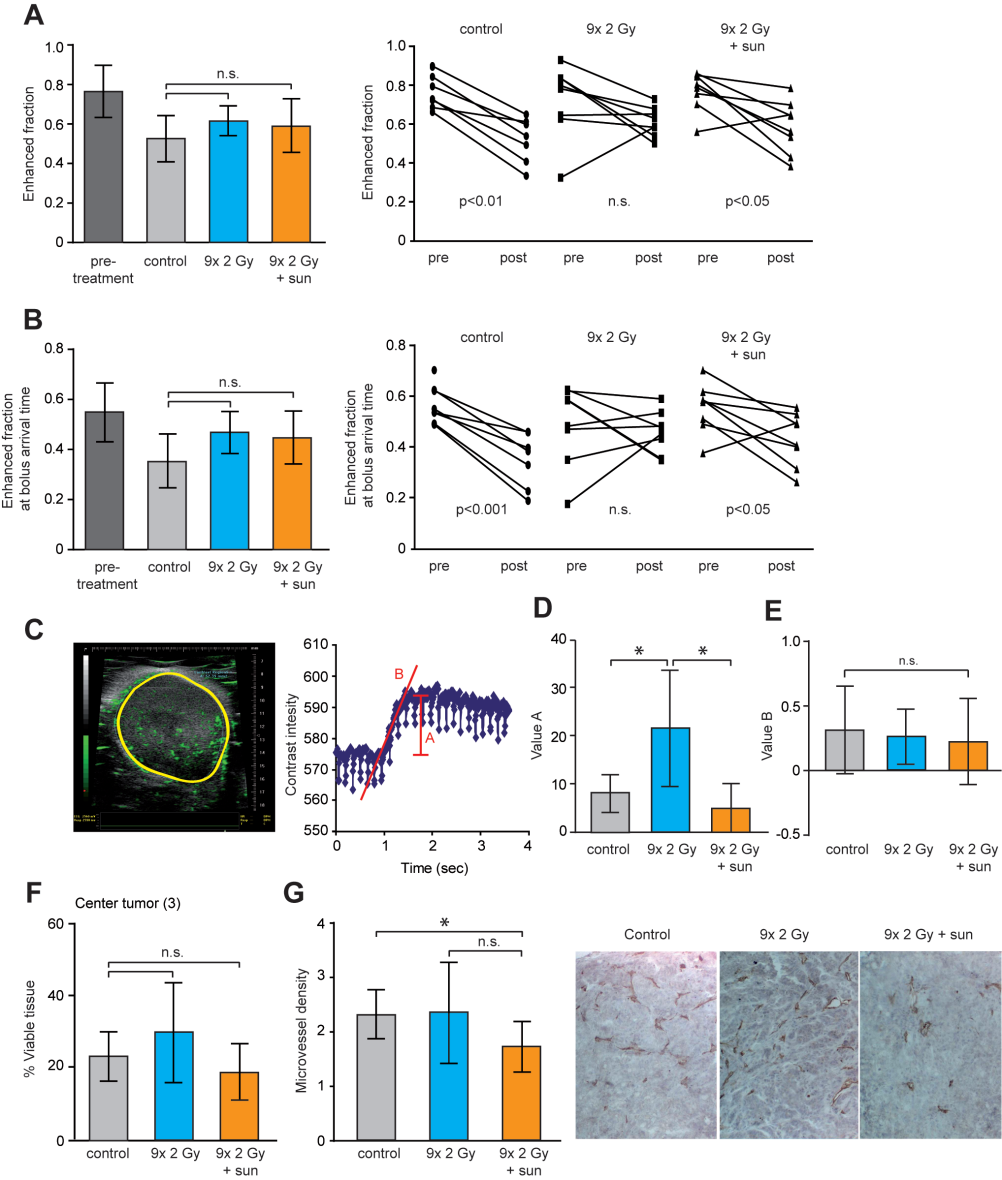
Enhanced tumor perfusion after RTx is counteracted by low dose sunitinib treatment HT29 xenografts in balb/c nude mice were grown to 100 mm^3^ and treated as indicated. RTx consisted of daily 2 Gy fractions (5/week). In case of combination therapy, sunitinib was daily administered by oral gavage (20 mg/kg/day) starting in the second week of RTx. DCE-MRI scans were performed for each tumor before treatment and after treatment. Controls did not receive any treatment. **A.** The left panel shows the enhanced fraction of voxels pretreatment and after each treatment. The right panel shows the matched pre- and post treatment measurements. **B.** Similar as in **A.** for the enhanced fraction of voxels at bolus arrival time. **C.** Representative image of a contrast-enhanced ultrasonography as performed on the tumors after treatment (left panel). The right panel shows the alterations in contrast intensity in the tumor over time. Parameter A represents tumor blood volume, and parameter B represents the velocity of the blood flow. **D.** Effect of treatment on tumor blood volume (parameter A) as determined by contrast-enhanced ultrasonography. **E.** Effect of treatment on tumor blood flow (parameter B) as determined by contrast-enhanced ultrasonography. **F.** Percentage of viable tissue in the center of the tumor as determined by H/E staining. Similar as for DCE-MRI analyses the area of interest was defined as 1/3 of the total tumor area that was located in the center of the tissue. **G.** The microvessel density within the viable tissue of the complete tumor was measured by IHC staining of CD31. All data are shown as average +/− SD N = 7-8 per experimental group. * *p* < 0.05 (Mann-Whitney U test).

Our initial experiments showed a benefit of low dose sunitinib in combination with RTx. Accordingly, we asked whether low dose sunitinib prevented the enhanced perfusion induced by RTx. Therefore, the effect of RTx in combination with low dose sunitinib (20 mg/kg/day) on tumor perfusion and cell viability was assessed. To allow analysis of changes in each individual tumor, DCE-MRI was performed prior to and after treatment for each tumor. While the follow-up time was too short to observe an effect on tumor growth (Supplementary Figure S5A) the enhanced fraction of voxels in the untreated tumors decreased, indicative of decreased tumor perfusion accompanying rapid tumor growth (Figure 5A). This reduction was counteracted by RTx^FR^. Combination of RTx^FR^ with sunitinib significantly reduced the fraction of enhanced voxels, similar to the non-treated tumors (Figure 5A). Comparable observations were made with the fraction of enhanced voxels at BAT (Figure 5B). Of note, the classical parameters Ktrans and Ve displayed similar trends but did not reach statistical significance (Supplementary Figure S5B+S5C). Furthermore, a more pronounced effect was observed in the center of the tumor for the fraction of enhanced voxels at BAT (Supplementary Figure S5D).

To strengthen the DCE-MRI observations, contrast enhanced ultrasound was performed in order to measure the velocity of the blood flow and the relative blood volume within the tumor (Figure 5C) [32]. A significant increase in tumor blood volume following RTx^FR^ was detected (Figure 5D). Combining RTx^FR^ with low dose sunitinib resulted in tumor blood volumes similar to the non-treated tumors (Figure 5D). To validate the functionality of the blood vessels, the velocity of the blood flow in the tumors was determined. No change after either RTx^FR^ or combination with low dose sunitinib was found (Figure 5E). Finally, while HE stainings did not reveal significant changes in the percentage of viable tissue in the center of the tumor, a significant decrease in microvessel density after sunitinib treatment was observed (Figure 5F and 5G). Collectively, these findings suggest that RTx might enhance tumor perfusion by induction of a pro-angiogenic tumor which can be counteracted by low dose sunitinib treatment.

## DISCUSSION

In the current study we explored the interaction between radiation therapy (RTx) and angiostatic drug treatment. Our results provide evidence that RTx can augment tumor perfusion. This is accompanied by decreased tumor hypoxia and results in tumor cell repopulation, mainly in the hypoxic center of the tumor. The response involved the induction of a pro-angiogenic response in tumor cells by RTx. The increased perfusion was found to be counteracted by low dose angiostatic drug treatment which might underlie the improved antitumor effect of combination therapy.

We and others have shown that angiostatic drugs can enhance the effect of RTx in preclinical studies [3, 17-19]. Part of this effect has been linked to vessel normalization which transiently improves tumor oxygenation during angiostatic therapy. The short window of normalization observed in mice, i.e. a few days [8, 9, 12, 33], suggests that it only plays a limited role during fractionated RTx regimes that last for several weeks. In addition, whether vessel normalization occurs in patients receiving angiostatic therapy as well as the duration of this normalization remains to be established. Moreover, angiostatic therapy can also be beneficial when given during or after RTx [5, 34]. The latter was confirmed in the current study further suggesting that mechanisms other than vessel normalization contribute to the interaction between RTx and angiostatic therapy. Here, we provide evidence for such an alternative mechanism. By applying DCE-MRI as a non-invasive method to monitor the tumor vascular function we found that RTx^FR^ enhanced tumor perfusion. This is in line with observations in patients [25-27, 35-37] and in different preclinical tumor models [30, 31, 38]. In the current study, we measured perfusion during the course of RTx^FR^ revealing that the induction of tumor perfusion became apparent after 2 weeks of treatment. Collectively, these results support the observation that RTx^FR^ can induce persistent changes in tumor perfusion in different tumor types. On the other hand, high dose RTx (either single dose or fractionated) has also been shown to cause vascular shutdown/hypoxia [28, 39, 40] indicating that the dose/fraction is a critical variable in predicting the vascular response to RTx. Of note, the high dose fractionated RTx in combination with anti-VEGF treatment did delay tumor growth more efficiently as compared to either treatment alone [28].

Detailed tissue analyses revealed that the enhanced perfusion was associated with a reduction in tumor hypoxia. In line with this, RTx has resulted in increased pO_2_ levels in different cancer models [41]. Previously, increased tumor oxygenation during RTx has been attributed to different mechanisms, i.e .i) decreased oxygen consumption, ii) increased inflammation, and iii) reduced tumor volume [42]. Our data demonstrate that increased tumor oxygenation might also result from enhanced perfusion.

The use of DCE-MRI scans with high spatial resolution allowed us to analyze perfusion in distinct regions of the tumor. While the rim of the tumor was always well perfused, a poorly perfused region was observed in the center of the tumor. Interestingly, it was in this center that we observed the most pronounced enhancement in perfusion. Rather than a beneficial effect on RTx efficacy, we observed that the improved perfusion was associated with repopulation of cancer cells and an increased number of tumor blood vessels. These findings are clinically relevant as repopulation of cancer cells during RTx^FR^ has been recognized as an important cause of treatment failure [43, 44]. However, it should be noted that the current study was confined to a single non-orthotopic tumor model. Thus, whether the response represents a tumor-type specific or more common response awaits future studies in different *in vivo* tumor models as well as in patients.

Further analysis of the mechanisms underlying the improved perfusion and vascularization identified induction of the pro-angiogenic molecules VEGF and PlGF, both *in vivo* and *in vitro*. This corroborates with previous studies in which RTx^SD^ and RTx^FR^ were shown to induce the expression of angiostimulatory factors including VEGF [18, 23, 24, 45, 46]. We now confirm in time-course experiments that a clinically relevant schedule of RTx^FR^ induces a potent angiogenesis response. Previously, RTx^FR^ in tumor bearing dogs did not increase the circulating levels of VEGF [47]. However, expression levels in the tumor were not determined suggesting that the local induction in the tumor tissue is not reflected systemically. Nevertheless, the increased expression of angiogenic factors like VEGF appears to be functionally relevant as inhibition of VEGF receptor signaling with sunitinib counteracted the increased perfusion and augmented the antitumor effect of RTx. This confirms previous observations where potentiation of either RTx^SD^ or RTx^FR^ by angiostatic therapy was observed in different tumor models [3, 18-22, 38]. Importantly, we now show that the beneficial effect is achieved with low dose angiostatic therapy, i.e. a dose that did not affect tumor growth rates when applied as monotherapy [48]. This supports our previous findings showing that concurrent scheduling of RTx^SD^ with sunitinib allowed dose reduction of sunitinib without affecting therapeutic efficacy [17]. In addition, it has been demonstrated in a xenograft glioblastoma model that the therapeutic effect of RTx (3x 5 Gy) improved when combined with low dose VEGF-Trap [49]. These findings indicate that the maximum effective dose of angiostatic drugs in combination with RTx is below the maximal tolerated dose. Comparable observations were made when combining angiostatic drugs with photodynamic therapy [12]. These observations are relevant as the clinical implementation of combination therapy with angiostatic drugs has been hampered due to the observed increase in the severity and frequency of side effects, including the occurrence of severe toxicities such as bowel perforations or hemorrhagic events [50, 51].

For the tumor perfusion analysis, we focused on conventional 2Gy RTx^FR^ as i) this is the most commonly applied clinical treatment schedule with curative intent and ii) because RTx^FR^ induced a more pronounced pro-angiogenic response. Nevertheless, tumor growth inhibition was also observed when low dose sunitinib was combined with single dose (5 Gy) RTx (5 Gy). It is known that single high dose of RTx can enhance endothelial cell apoptosis and decrease tumor perfusion shortly after RTx (6-72h) [39, 40, 52]. In correspondence to this, we did not observe enhanced tumor perfusion 1 week after RTx^SD^. However, it has also been described previously that RTx^SD^ of 5 Gy can increases pO_2_ and tumor perfusion 14 days after RTx [53, 54]. We did observe that low dose sunitinib started to deflect the tumor growth curve 2 weeks after RTx^SD^ of 5 Gy, suggesting that the increased perfusion and subsequent tumor growth at this time point was inhibited. These data further exemplify that it is important to interpret the tumor perfusion depending on the dose and schedule of the RTx and the time point of perfusion measurement. Together, these results indicate that patients with advanced cancer who often undergo palliative single dose RTx for reduction of pain or other symptoms may significantly benefit from the addition of low dose angiostatic treatment in this setting.

In summary, we set out to investigate the effects of combining clinically relevant schedules of RTx with the angiostatic drug sunitinib on tumor growth and tumor perfusion. While the commonality of these observations awaits further confirmation in different tumor models as well as in cancer patients, our data suggest that irradiation induces a proangiogenic response in tumors cells which could render the tumor more sensitive to low dose sunitinib. These observations are especially important for the potential translation of this combination therapy to the clinical setting as it could reduce toxicities. Finally, while we focused on RTx^FR^ schedules, our results also demonstrate benefit of low dose sunitinib treatment after RTx^SD^. This observation could be well translated to large patient groups receiving palliative RTx.

## MATERIALS AND METHODS

### Human umbilical cord endothelial cells (HUVEC) isolation

Primary HUVEC were isolated from human umbilical cords. The vein was flushed with sterile PBS), filled with trypsin and incubated 15 minutes at 37°C. The vein was flushed with RPMI + 10% FCS + 10% human serum (HS) and cell suspension was collected. Cells were centrifuged for 5 minutes at 250 rcf. After aspirating the medium, the cells were resuspended in complete RPMI and seeded in a 0.2% gelatin coated T25 flask. HUVECs were washed with PBS 2 and 24 hours after isolation to remove the remaining red blood cells. HUVECs were maintained up to passage 4.

### Cell culture

HUVECs were cultured in (hereafter complete) RPMI + 10% FCS + 10% HS + 1% Penicillin Streptomycin + 1% L-glutamine, in 0.2% gelatin coated flasks. Endothelial cells were passaged 1:3 every 3-5 days. Tumor cell lines (HT29 colon carcinoma and D384 glioblastoma) were cultured in DMEM + 10% FCS + 1% Penicillin Streptomycin + 1% L-glutamine, and passaged 1:10 every 3 days. Incubation was at 37°C, with 5% CO_2_ in humified air. Cell lines were authenticated by STR profiling (BaseClear, Leiden, The Netherlands) and were repeatedly found negative for mycoplasm infection as checked by PCR. During fractionated irradiation experiments, cells did not require passaging and at the end of each week, culture medium was collected and centrifuged for subsequent analyses (tumor conditioned medium).

### Endothelial cell migration assay

In a 0.2% gelatin coated 96-well Costar clear plate, 1×10^4^ HUVECs were seeded in each well in 100 μL complete RPMI. Cells were grown into a confluent monolayer, and starved overnight with 100 μL RPMI + 2% HS. Next day, cells were scratched using a 96-well pintool. After washing the cells twice with PBS, the tumor conditioned medium (1:1 with RPMI + 2% HS) or compound was added. Each condition was performed in triplicate with 3 different HUVEC isolations. Pictures were taken at *t* = 0 and *t* = 7h. The pictures were analyzed with ImageJ, measuring the area of the scratch.

### Endothelial sprouting assay

This assay was performed as described previously [55]. In short, HUVECs (4×10^4^ cells/mL, passage 1 or 2) were resuspended in 20% metocellulose, 10% HS and 70% RPMI, and hanging drops of 25μLwere incubated for 16h. Next day, 30 spheroids per condition were embedded in 200 μL growth factor reduced Matrigel (BD bioscience) in a 24-well plate. The tumor conditioned medium (1:1 with RPMI + 2% HS) or compound was added, in a total volume of 500 μL. After 24h pictures were taken. Of each condition, 20 spheroids were analyzed, measuring the number of sprouts. A sprout was defined as tubular structure extending from the spheroid into the surrounding matrix while still being connected to the spheroid. Experiments were performed with three different HUVEC isolations.

### RNA isolation, cDNA synthesis and qPCR

Isolation of RNA from cultured cells was performed using the RNeasy kit (QIAgen). For RNA isolation from the mouse xenografts the mirVANA kit (Life Technologies) was used, excluding the purifying miRNA step. The final RNA concentration was determined using the Nanodrop ND-1000. Subsequent reverse transcription was performed using 1 μg RNA, with the iScript kit (Biorad) following the manufacturer's protocol. The resulting cDNA was used for the qPCR reaction, using the SYBR green supermix (Biorad) with a total sample volume of 25 μL. For primers sequences, see Supplementary Table 1. With the CFX96 (Biorad) the following cycling conditions were used: 95°C for 5 min, followed by 95°C for 10 sec and 60°C for 30 seconds for 40 cycles. Expression levels were normalized to 4 reference genes, i.e. beta-actin (ACTB), peptidylprolyl isomerase A (PPIA), Hypoxanthine-guanine phosphoribosyltransferase (HPRT), and beta-2 microglobulin (B2M), as described previously [56].

### ELISA

Enzyme-linked immunosorbent assays for human VEGFA was performed (ELH VEGF-001, RayBiotech) according to the manufacturer's instructions, using supernatant of the *in vitro* cultured cancer cells. Expression levels were normalized to the number of cells.

### Mouse xenograft studies

Mice were housed at the Radiation Research Institute, Churchill Hospital, Oxford, UK. All procedures were carried out under a Home Office license [PPL: 30/2922]. HT29 cells were detached with trypsin, then washed in PBS twice and mixed in 1:1 in serum-free DMEM medium and Matrigel before inoculation in mice. Five million cells in 100 μL Matrigel/ DMEM suspension were injected subcutaneously in the lower right flank of 6- to 7-week-old female BALB/c nude mice. Tumor growth was monitored 3-4 times per week measuring the length (L), width (W), and height (H) of each tumor with calipers. Volumes were calculated from the formula 1/6*π*L*W*H. The mice were randomized into the experimental groups, aiming for equal average tumor size in each group. For selected treatment groups, 200 μL sunitinib (2mg/ml in 5%DMSO/H_2_O corresponding to 20mg/kg/day) was administered daily with oral gavage, 4 hours after irradiation. Control groups did not receive any treatment. One hour before being sacrificed, mice were injected intra-peritoneally (i.p.) with 1.5 mg of pimonidazole (hypoxyprobe-1; Chemicon International). Next, mice were sacrificed by intravenous (i.v.) injection of pentobarbital. Tumors were harvested and fixated for further analysis. In line with animal welfare regulations tumor imaging and irradiation could not be performed on the same day. Thus, on the last day of the treatment week, tumor tissues (including those that were not imaged) were collected before application of the 5th, 10th or 15th dose, respectively.

### Irradiation

Cultured cells and eggs received the desired dose of γ-radiation using a ^60^Co source (Gammacell 200; Atomic Energy of Canada, Mississauga, Ontario, Canada).

Mice were irradiated using Xstrahl RS320 X-Ray irradiator (Xstrahl Ltd. UK). Mice first received100 μL i.p. anesthetics 1:1:8 hypnorm: hypnovel: sterile water and were then lead-shielded, so that only the tumor was exposed to irradiation.

### Hematoxylin/eosin staining and immunohistochemistry

Hematoxylin/eosin (H/E) and immunohistochemical (IHC) stainings were performed on 4 μm thick paraffin sections of mouse xenograft tumors. Following deparaffinization in xylene, the slides were rehydrated through a graded series of alcohol. For the H/E staining, the slides were emerged in haematoxylin for 3 minutes. After washing thouroghly, the slides were stained with eosin for 10 seconds and again washed with water. Immunohistochemical (IHC) staining was performed on 4 μm thick paraffin sections of mouse xenograft tumors. Following deparaffinization in xylene and rehydration through a graded series of alcohol, endogenous peroxidase activity was blocked by 20 minute incubation in 0.3% H_2_O_2_/PBS. Next, antigen retrieval was performed in sodium citrate solution (pH 6.0) using a pressure cooker. After a blocking step with 5% BSA/PBS at room temperature (RT), the samples were incubated for 1 hour at RT or at 4°C overnight with the primary antibody diluted in 0.5% BSA/PBS. Control slides were incubated with 5% BSA/PBS. Following primary antibodies were used: pimonidazole (hypoxyprobe-1; Chemicon International; 1:50), CAIX (M75, 1:50), CD31 (SZ31, Dianova), and Ki-67 (M7240; Dako; 1:50). Next, the slides were incubated for 30 minutes at RT with the appropriate secondary biotinylated antibody, followed by incubation with strep-ABC-HRP for 30 minutes at RT (1 uL avadin and 1 uL biotin in 500 uL PBS). Finally, staining was visualized with 3,3-diamino-benzidine-tetra hydrochloride (DAB), 0.3 mg/mL in 1 mL PBS. All slides were counterstained with hematoxylin and mounted in Entellan (Merck) for microscopy. Pictures of the entire tumor section were taken at 40x or 100x magnification, and analyzed quantitatively in ImageJ using color deconvolution as described previously [57]. For analysis of viable and necrotic tissue, the H/E-staining was used. The percentage of hematoxylin stained tissue, considered as viable tissue, relative to the eosin stained tissue was calculated using image J. For CD31 staining, the number of vessels in the viable tissue was counted, where a vessel was considered to be at least 50 pixels. For the KI67 quantification, the number of DAB-positive nuclei was counted, and compared to the total number of nuclei in the viable tissue. For CAIX and pimonidazole, the area of DAB-positive tissue was quantified.

### Dynamic contrast enhanced magnetic resonance imaging

Anesthesia was induced and maintained with isoflurane (1-4% in air) so as to maintain a respiration rate of 40-60 breaths per minute, and temperature was maintained at 35°C using a homeothermic temperature maintenance systems [58]. MRI was performed at 4.7 and 7.0 T (Varian, VNMRS console) using 25 mm id birdcage coils (Rapid Biomedical, Germany). Dynamic contrast enhanced MRI (DCE-MRI) was performed using a respiratory-gated 3D gradient echo scan (TE = 0.6 ms, TR = 1.15 ms, nominal 5 degree flip angle) with an isotropic resolution of ca. 420 micron and a respiratory rate dependent frame acquisition time of ca. 8-10 seconds. Fifty frames were acquired with a bolus of Gadolinium (Gd) solution (25 ul, Omniscan GE HEALTHCARE) infused automatically by syringe pump (PHD2000, Harvard Apparatus) over 5 seconds starting at the beginning of frame 11. RF field in homogeneities were accounted for using a respiratory-gated implementation of the Actual Flip Angle technique [59] and baseline T1 was measured using a variable flip angle [60] sequence based upon the scan frame described above. For the analysis the tumor was firstly segmented manually from the average image of the DCE sequence using ITK-SNAP [61]. The MR signal was converted to Gd concentration using the method described previously [62]. Non-enhancing voxels where defined as voxels in which the MR signal did not exceed 3 standard deviations of the pre-injection baseline signal during the experiment. The time at which a voxel began to enhance, commonly referred to as the bolus arrival time (BAT), was determined using a piece-wise linear fit to the Gd *vs* time curve [63]. The initial area under the Gd curve (iAUC) was measured as an indicator of perfusion. In this case the first 150 seconds after injection were integrated. A population averaged arterial input function (AIF) was assumed for pharmacokinetic modeling of the DCE data based on the data described previously [64]. To define the different regions in the tumor, the rim (area 1) was calculated 3 pixels inwards from the identified tumor edge. The remaining volume of the tumor was divided as the outer region (2/3 of the volume) and the center (1/3 of the volume) of the tumor.

### Contrast-enhanced micro-bubble ultrasono-graphy

Tumor perfusion was measured with the Vevo 770 system as described [65], 24h after the last dose of RTx and sunitinib. Mice were anaesthetized with isoflurane gas (1-4% in air) and prepared for the ultrasound with a tail vein cannula. Body temperature was maintained with a heat-mat. Coupling gel was applied over the tumor and the transducer was calibrated in the middle of the tumor. Next, baseline images of the whole tumor (a loop) were acquired before injection of the micro-bubble contrast-enhancement injection. After injection of 60 μL of VEVO microMarker visual sonics in the tail vein, a second loop of images for contrast-enhancement were acquired. For analysis, the region of interest (ROI) was selected for each image manually. The base line loop was compared with the contrast loop, using Vevo 770 contrast mode software (Visualsonics).

### Statistical analysis

For the *in vitro* functional assays and gene expression analyses, the means of each independent experiment were used for statistical analysis with the Mann-Whitney U test, which was performed using SPSS 20.0.0. Regarding the *in vivo* experiments, for tumor growth analysis a one-way-ANOVA was used, with a Bonferroni's multiple comparison test. Other analyses were performed with the two-tailed Student's *t*-tests unless indicated otherwise. A p-value ≤ 0.05 was considered as a statistical significant difference.

## SUPPLEMENTARY MATERIALS TABLE AND FIGURES


