# Supplementary material for: Low dose angiostatic treatment counteracts radiotherapy-induced tumor perfusion and enhances the anti-tumor effect
Source: Oncotarget. 2016 Oct 21;7(47):76613–27. doi: 10.18632/oncotarget.12814 (PMC5363534; doi:10.18632/oncotarget.12814)
Supplement: Supplementary file 1 [file oncotarget-07-76613-s001.pdf]

Low dose angiostatic treatment counteracts radiotherapy-induced tumor perfusion and enhances the anti-tumor effect

Supplementary Material

Supplementary Table S1. Primer sequences for qPCR

Gene	Forward primer (5'-3')	Reverse primer (5'-3')
ACTB	TTCCTATGTGGGCGACGAG	TCCTCGGGAGCCACACG
HPRT	TGCTGAGGATTTGGAAAGG	TCACATCTCGAGCAAGACGT
PPIA	AGCATGTGGTGTTTGGCAAA	TCGAGTTGTCCACAGTCAGC
B2M	TCCATCCGACATTGAAGTTG	ACACGGCAGGCATACTCAT
VEGFA	CCATCGACAGAACAGTCCTT	CGAATCCAATTCCAAGAGGG
GAL1	TGCAACAGCAAGGACGGC	CACCTCTGCAACACTTCCA
EGF	GTGACTCTGAATGTCCCCTG	CACCACTTCAGGTCTCGGTA
PDGF	GCGAGCTGGAGAGCTTGG	GGTGCGGTCTATGAGGCG
PlGF	TGCAGCTCCTAAAGATCCGT	GGGAACAGCATCGCCGCA
ANG1	AGCTACCACCAACAACAGTG	GCAAAGATTGACAAGGTTGTGG

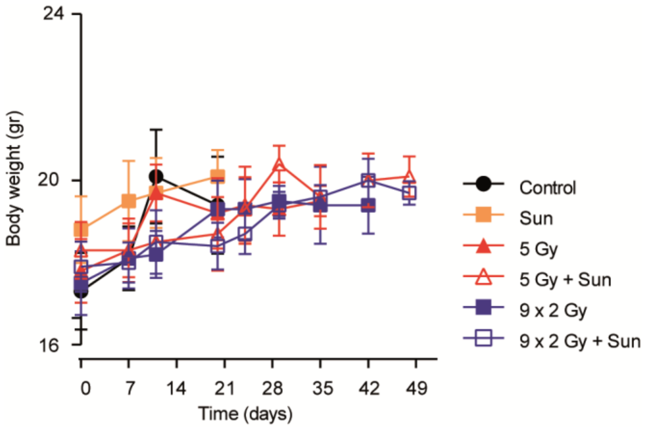

Supplementary Figure 1. Toxicity study in balb/c nude mice

Body weight of mice treated with I) single dose RTx of 5 Gy, II) 5 fractions of 2 Gy per week (total of 9 fractions), III) sunitinib 20 mg/kg/day, IV) 5 Gy plus sunitinib 20mg/kg/day, VI) 9x 2 Gy plus sunitinib 20/mg/kg/day. Sunitinib was started after the first dose of RTx and continued until the end of the experiment. Data are shown as average +/- SEM.

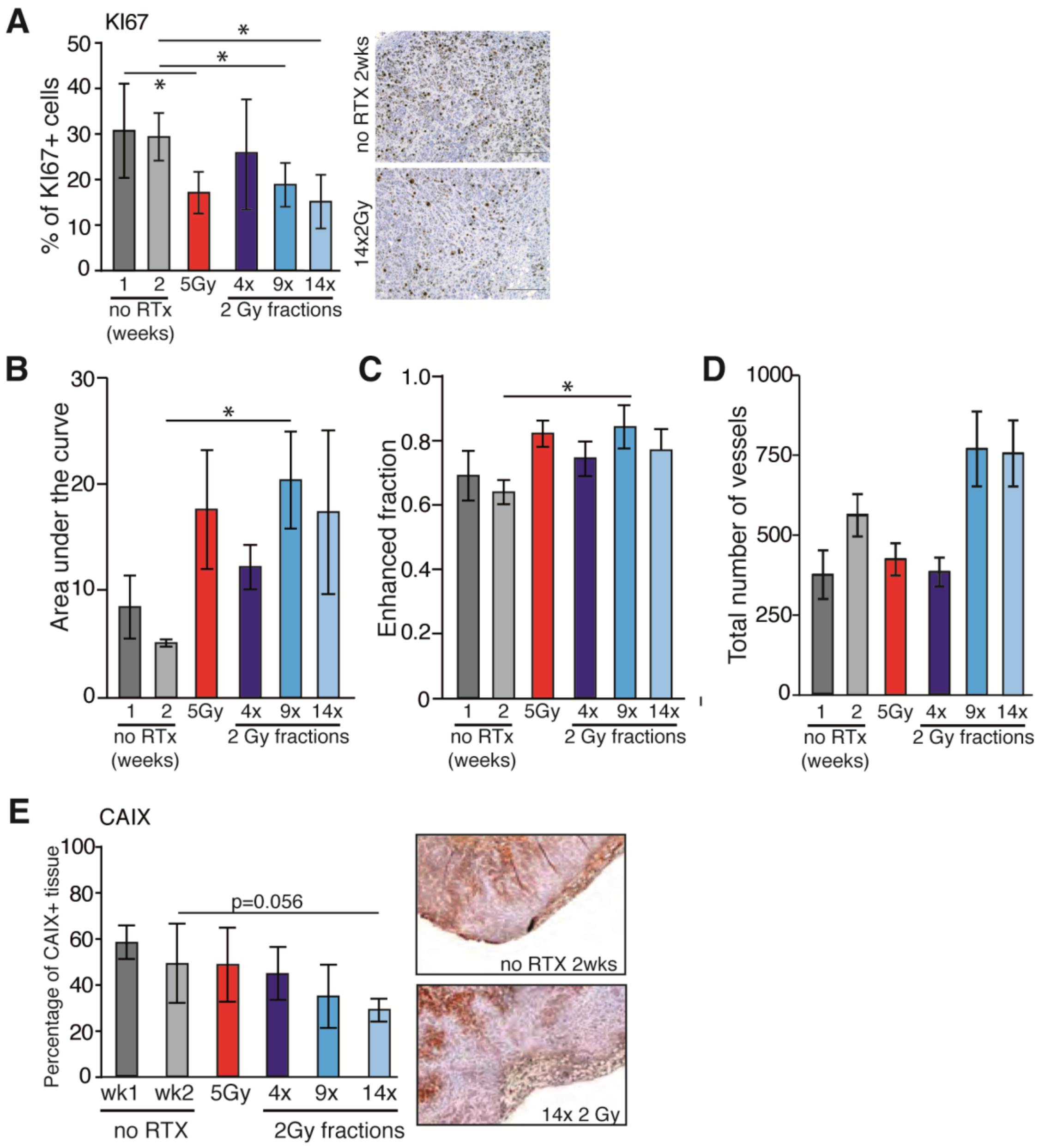

Supplementary Figure 2. Tumor cell proliferation, apoptosis and hypoxia during RTx. (A) The proliferation index of the HT29 tumors was determined by IHC staining of KI67 (brown). (B) The initial area under the curve (iAUC) of each tumor as determined by DCE-MRI. (C) The fraction of enhanced voxels during the scan as determined by DCE-MRI. (D) Total number of blood vessels in the viable tissue was measured with IHC staining of CD31 (dark brown). (E) Tumor hypoxia of HT29 xenografts in mice was determined after RTx, with IHC staining for the hypoxia marker CAIX. Data are shown as average +/- SD. N=4-5 per experimental group. * $p < 0.05$ (Mann-Whitney U test).

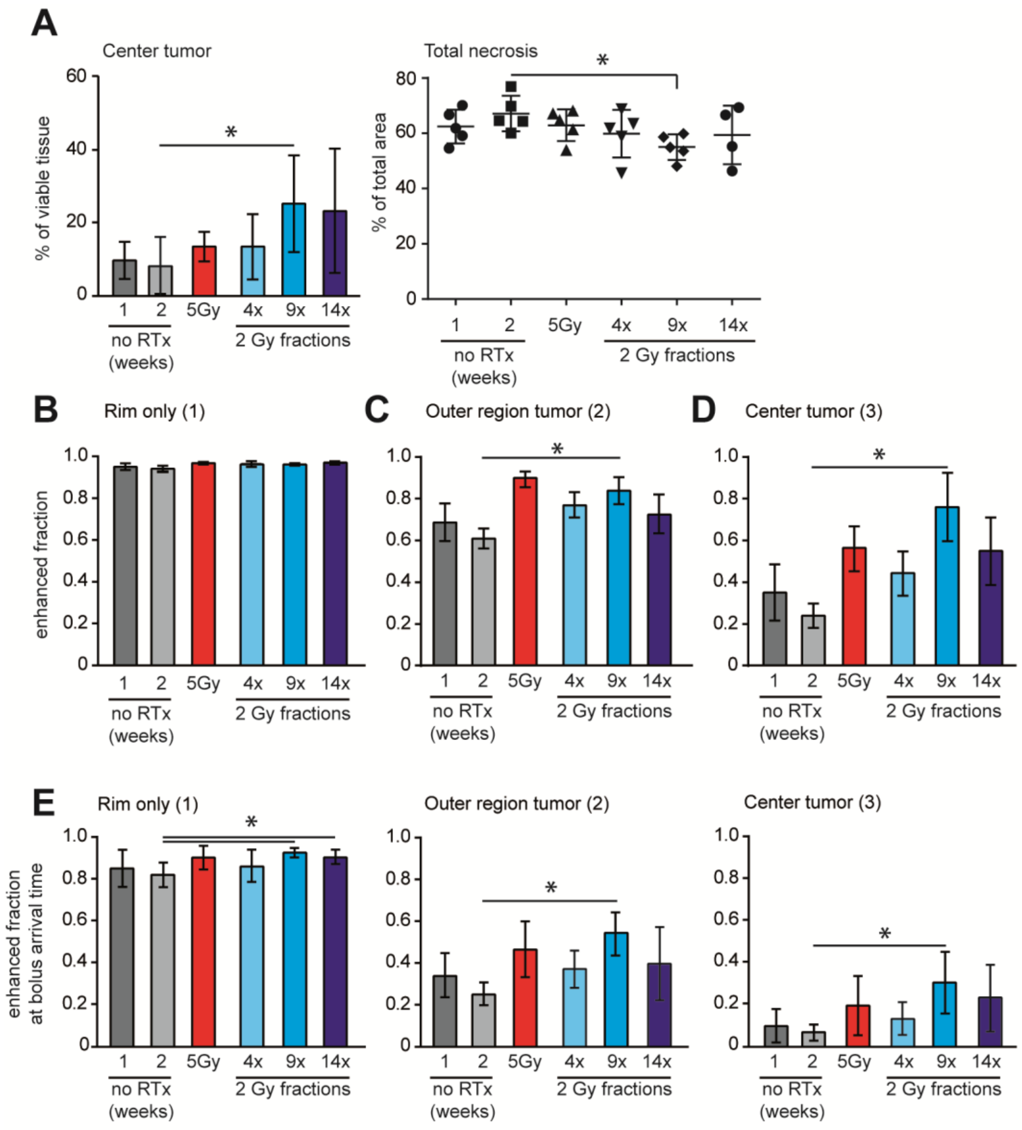

Supplementary Figure 3. Effect of RTx on tissue viability and perfusion in different regions of the tumor.

(A) The percentage of viable tissue in the center of the tumor (left panel) and the percentage of necrotic tissue in the entire tumor tissue (right panel) as determined with H/E staining. (B-D) Enhanced fraction of voxels in the three different regions of the tumor as determined by DCE-MRI. (E) Enhanced fraction of voxels at bolus arrival time for the tree different regions of the tumor as determined by DCE-MRI. All DCE-MRI data are shown as average +/- SD. N= 4-5 per experimental group. * $p < 0.05$ (Mann-Whitney U test).

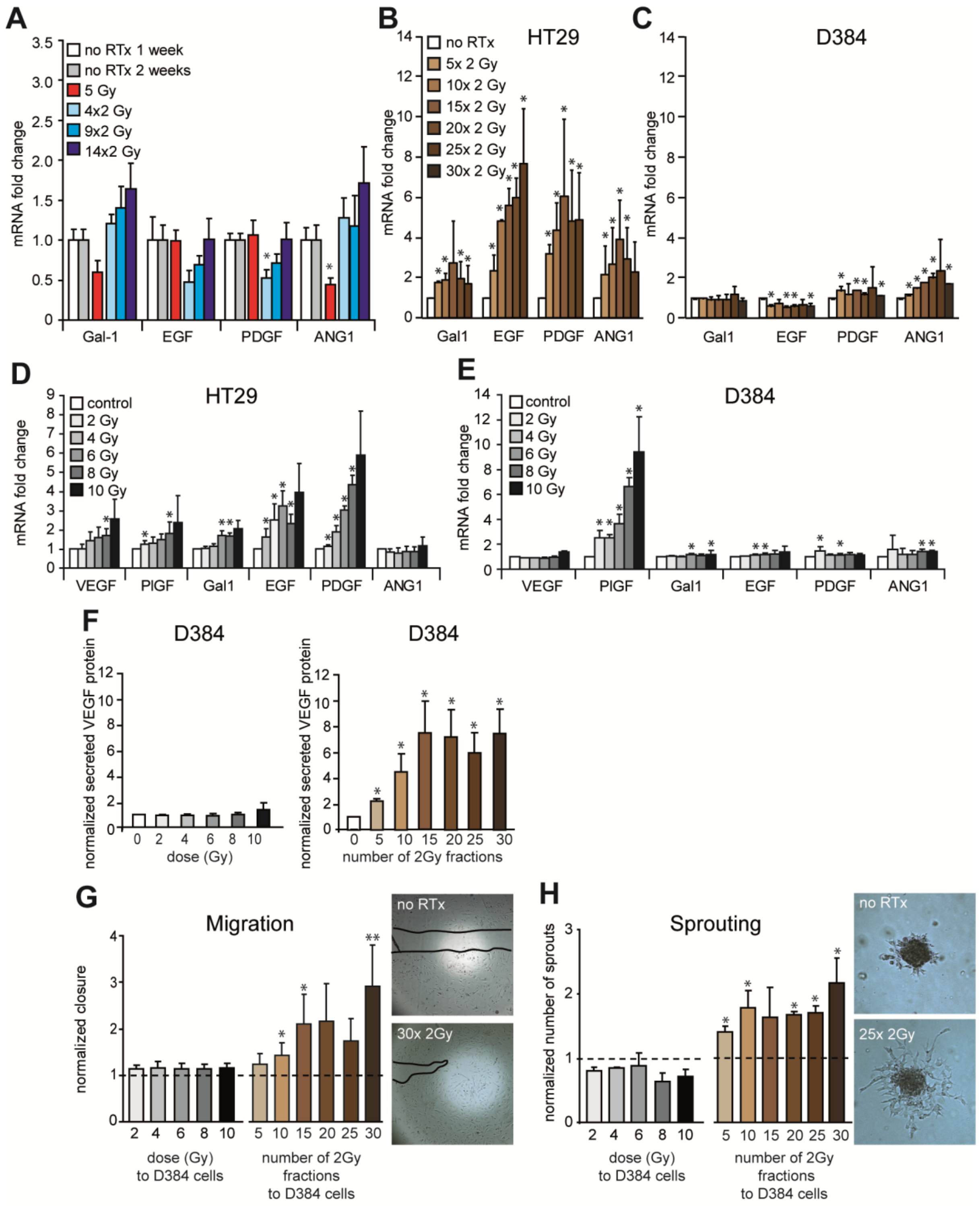

Supplementary Figure 4. Pro-angiogenic growth factor expression in vivo and in vitro after RTx in cancer cells

(A) Relative mRNA expression of pro-angiogenic growth factors in HT29 xenograft tumors in balb/c nude mice after single dose (5 Gy) or fractionated RTx. N= 4-5 per experimental

group. Gal-1 = galectin-1; EGF = epidermal growth factor; PDGF = platelet derived growth factor; ANG1 = angiopoietin-1. (B) Relative mRNA expression of pro-angiogenic growth factors in HT29 cells in vitro after different fractions of 2 Gy RTx. N=3 independent experiments; Gal-1 = galectin-1; EGF = epidermal growth factor; PDGF = platelet derived growth factor; ANG1 = angiopoietin-1; FGF = basic fibroblast growth factor. (C) Similar as in (B) but now for irradiated D384 cells. N=3. (D) Similar as (B) but now for single dose irradiated HT29 cells. N=3. (E) Similar as in (D) but now for D384 cells. N=3. (F) Normalized secreted VEGF protein expression in the supernatant of D384 cells after single dose or fractionated RTx. The protein expression was normalized to the number of cells. N=3. (G) Migration assay with endothelial cell (HUVEC) spheroids with conditioned medium of irradiated D384 cells. The width of the scratch was normalized to non-irradiated condition. N= 2-3 individual HUVEC batches for each batch of conditioned medium (N=3). (H) Sprouting assay with endothelial cell (HUVEC) spheroids with conditioned medium of D384 cells. The number of sprouts was normalized to non-irradiated condition. N= 2-3 individual HUVEC batches for each batch of conditioned medium (N=3). All DCE-MRI data are shown as average +/- SEM. * $p < 0.05$ (Mann-Whitney U test).

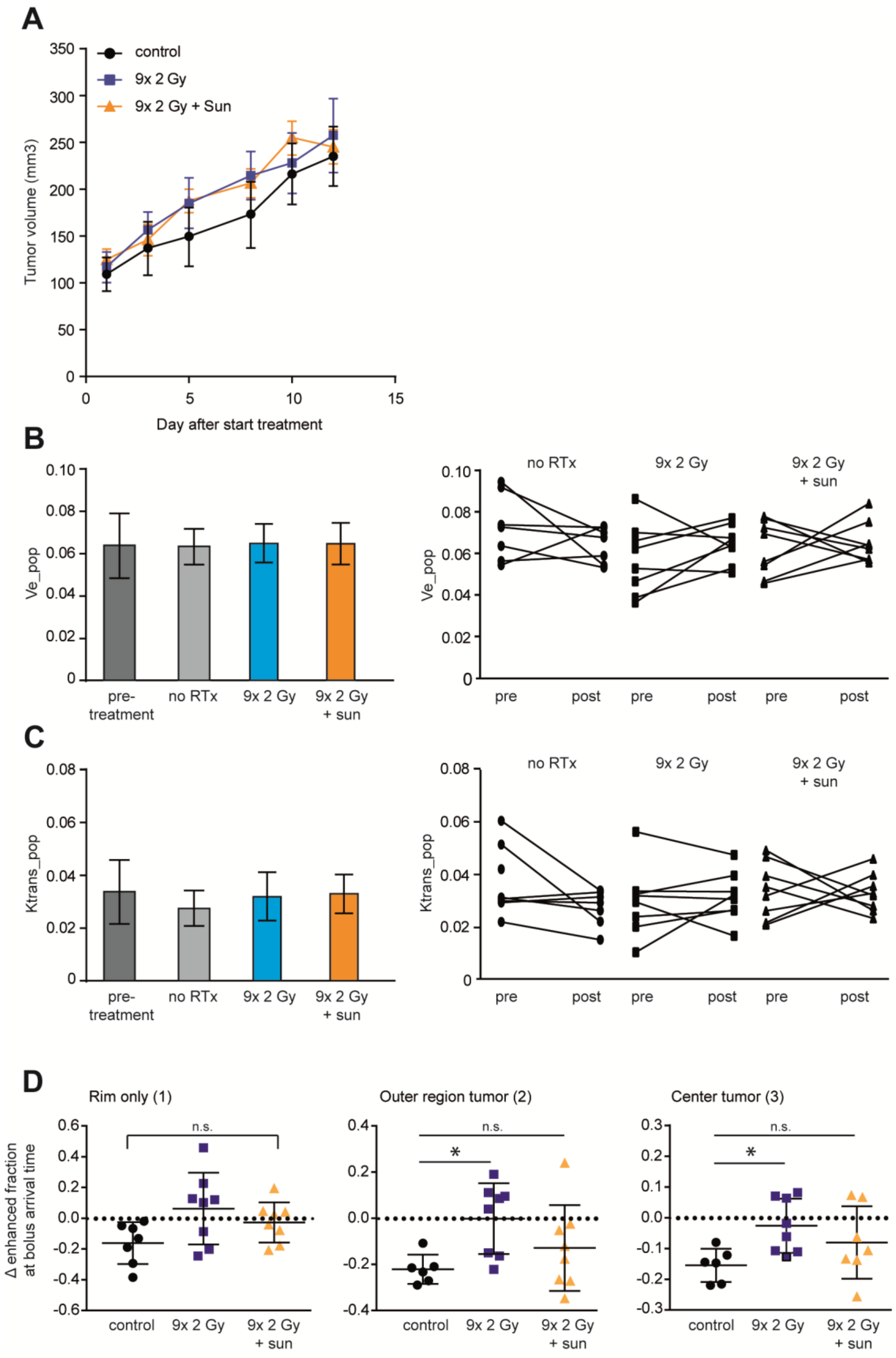

Supplementary Figure 5. HT29 xenografts treated with fractionated RTx with or without low dose sunitinib.

(A) Established HT29 tumor xenografts were grown to +/- 100mm^3 and then treated with fractionated RTx (9x 2 Gy) or fractionated RTx plus low dose sunitinib (20mg/kg/day). (B) DCE-MRI scans were performed for each tumor before treatment and after treatment. Ve was measured at each time point separately (left) and matched pre- and post treatment measurements are displayed in the right graph. (C) DCE-MRI scans were performed for each tumor before treatment and after treatment. Ktrans was measured at each time point separately (left) and matched pre- and post treatment measurements are displayed in the right graph. (D) Difference in enhanced fraction of voxels at bolus arrival time for the three different regions of the tumor. Difference in enhanced fraction is before and after treatment. * $p < 0.05$ (Mann-Whitney U test).
